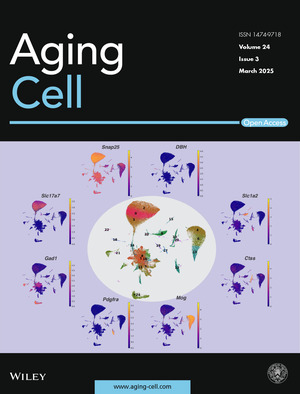# Additional Cover

**DOI:** 10.1111/acel.70046

**Published:** 2025-03-11

**Authors:** Akash Kumar Singh, Ila Joshi, Neeharika M. N. Reddy, Sushmitha S. Purushotham, M. Eswaramoorthy, Madavan Vasudevan, Sourav Banerjee, James P. Clement, Tapas K. Kundu, Tamunotonye Omoluabi, Zia Hasan, Jessie E. Piche, Abeni R. S. Flynn, Jules J. E. Doré, Susan G. Walling, Andrew C. W. Weeks, Touati Benoukraf, Qi Yuan

## Abstract

The cover image is based on the article *Locus coeruleus vulnerability to tau hyperphosphorylation in a rat model* by Tamunotonye Omoluabi et al., https://doi.org/10.1111/acel.14405